# Delayed introduction, transmission variation and susceptible dynamics explain spatial asynchrony during Republic of Korea's large pertussis outbreak

**DOI:** 10.1098/rsif.2026.0205

**Published:** 2026-10-21

**Authors:** Sang Woo Park

**Affiliations:** ^1^ School of Biological Sciences, Seoul National University, Seoul, Republic of Korea; ^2^ Institute for Data Innovation in Science, Seoul National University, Seoul, Republic of Korea

**Keywords:** pertussis, spatial synchrony, mathematical model, infectious disease dynamics, epidemiology, Bayesian inference

## Abstract

Infectious disease outbreaks typically exhibit a large-scale spatial synchrony, reflecting coupling through shared environmental forcing and human mobility. Exceptions to this theoretical expectation have been reported, but the conditions under which synchrony breaks down remain poorly understood. Republic of Korea's 2024–2025 pertussis outbreak provides a striking example, during which epidemic trajectories diverged substantially throughout the country: notably, the decline in spatial synchrony with distance was more pronounced than that reported in many previous outbreaks. Integrating high-resolution surveillance data from 252 municipalities and a Bayesian transmission model, we show that heterogeneity in introduction timing and differences in local transmission levels, which may reflect differences in either contact patterns or susceptibility, can drive such fine-scale asynchrony even when all locations share identical seasonal forcing for transmission rates. In particular, regional variation in baseline transmission levels can drive differential levels of susceptible depletion, thus shaping the potential for multiple epidemic waves. This analysis offers a unique perspective on spatio-temporal epidemic dynamics that depart from classical epidemic theory.

## 1. Introduction

Spatio-temporal variation in epidemic dynamics offers a powerful means for understanding factors that determine the timing and magnitude of epidemic waves across populations [1–4]. Ecological theory predicts that spatial coupling—via shared environmental forcing and human mobility—can synchronize epidemic dynamics between neighbouring locations, where epidemic waves propagate through space [5–7]. Consistent with this theory, large-scale spatial synchrony has been observed across diverse pathogens, including measles [7], dengue [8], influenza [9], respiratory syncytial virus (RSV) [4] and SARS-CoV-2 [10], highlighting the near-ubiquity of spatial coupling in shaping epidemic dynamics. However, an exception to this pattern has been observed in prevaccination pertussis epidemics, which exhibited substantially weaker spatial correlation than measles epidemics reflecting a stronger sensitivity to stochasticity and transients [11].

Pertussis is a vaccine-preventable respiratory disease caused by the bacterium *Bordetella pertussis* [12]. Following the introduction of diphtheria, tetanus and whole-cell pertussis vaccine in the mid-1950s in Republic of Korea and the later replacement with diphtheria, tetanus and acellular pertussis (DTaP) vaccine in 1989, the number of pertussis cases in Republic of Korea decreased dramatically, with fewer than 10 cases reported in 1995 compared to >10000 cases reported annually around 1955–1963 [13–17]. The current immunization schedule in Republic of Korea recommends DTaP vaccines at two, four and six months, with boosters at 15–18 months and 4–6 years, followed by tetanus toxoid, reduced diphtheria toxoid and acellular pertussis (Tdap) at 11–12 years and tetanus–diphtheria/Tdap boosters every 10 years thereafter [18]. However, pertussis cases have resurged in Republic of Korea and many other countries despite high vaccine coverage, raising concerns about the possibility of vaccine waning [19,20] and pathogen evolution [21–23]. Between 2024 and 2025, an unprecedented large pertussis outbreak occurred in Republic of Korea, resulting in more than 50 000 cases by early 2025 [24]. This outbreak marked a dramatic increase in the disease compared to 292 cases reported in 2023 and 21 cases reported in 2022, despite high vaccine coverage (91.1% of identified 2024 cases had a known vaccination history) [24,25]. Considerable spatio-temporal variation in disease burden has been observed during the outbreak, but we currently have limited understanding of the underlying spatio-temporal variation in epidemic dynamics [24]. Interestingly, this outbreak exhibited a pronounced spatial asynchrony in terms of both the size and timing of epidemic waves, with some municipalities experiencing only one of the two epidemic waves. This pattern deviates from theoretical expectations based on spatial coupling, raising questions about mechanisms driving this fine-scale spatio-temporal heterogeneity in pertussis epidemic (figure 1).

**Figure 1.**
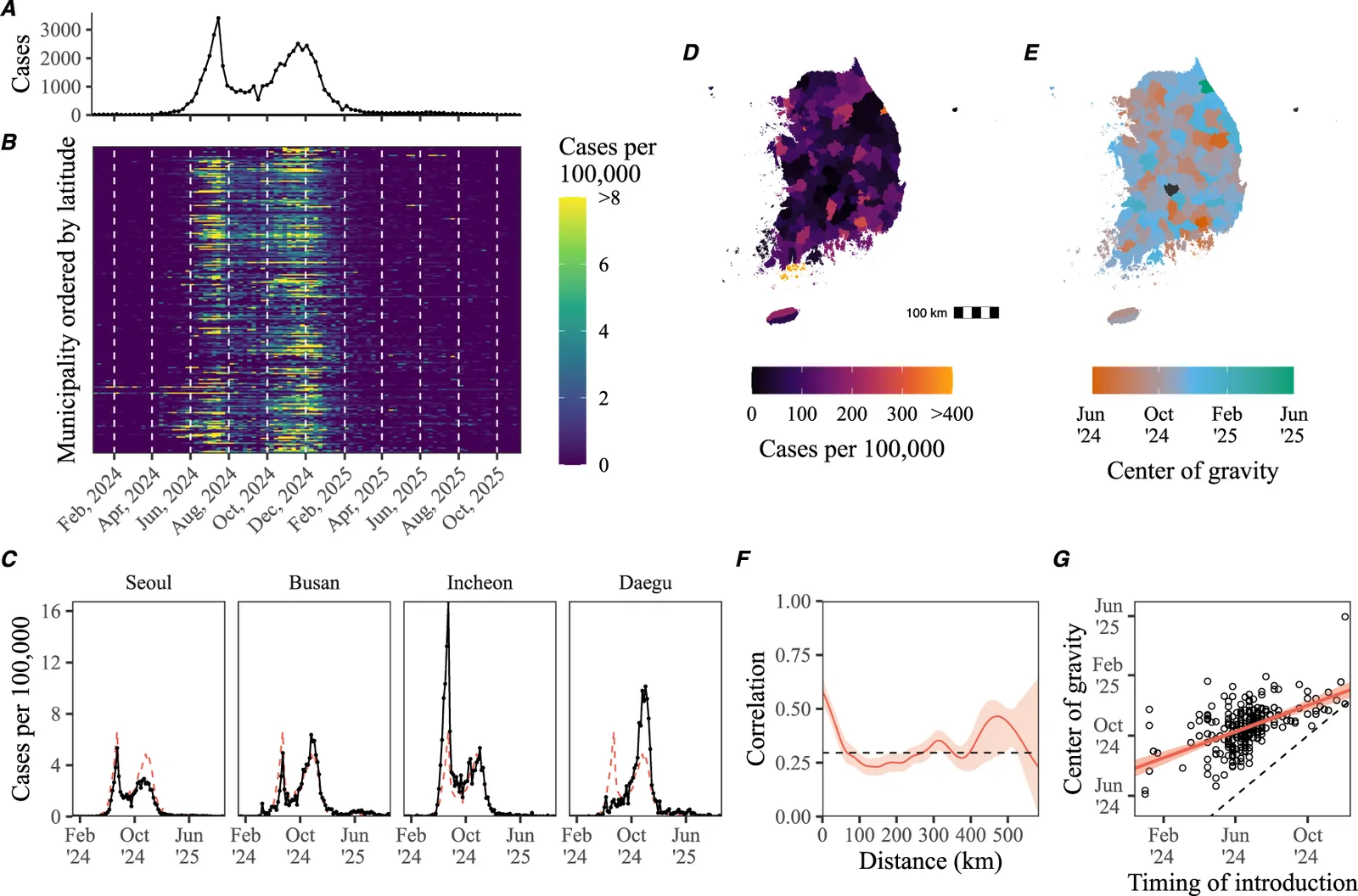
Spatio-temporal dynamics of pertussis outbreak in Republic of Korea, 2024–2025. (*A*) Weekly number of total reported cases in Republic of Korea. (*B*) Weekly number of reported cases per 100 000 individuals by municipality, ordered by latitude. (*C*) Weekly number of reported cases per 100 000 individuals in the four most populated cities are shown by black lines. Red dashed lines show the weekly number of total reported cases nationally. (*D*) Map of pertussis cases per 100 000 individuals between January 2024 and November 2025. (*E*) Map of centre of gravity, which represents the mean timing of cases. (*F*) Spatial synchrony in logged cases as a function of distance. The red solid line and shaded regions represent the median estimate and the corresponding 95% confidence interval. The dashed horizontal line represents the mean correlation. (*G*) Relationship between centre of gravity and the timing of introduction, defined as the first week when the number of reported cases is greater than 1 in 100 000. The red solid line and shaded regions represent the linear regression fit and the corresponding 95% confidence interval. The dashed line represents the one-to-one relationship.

To understand mechanisms driving the apparent spatial asynchrony during the large pertussis outbreak in Republic of Korea, we combine detailed spatio-temporal surveillance data from 252 municipalities with mathematical modelling approaches. We begin by showing that asynchrony in pertussis epidemics, captured by heterogeneous epidemic timing, is associated with differences in introduction delays. By contrast, the analyses of effective reproduction number reveal synchrony in underlying transmission patterns, suggesting that spatial asynchrony can arise despite synchrony in transmission. To test this hypothesis, we develop a spatio-temporal transmission model that allows for a shared transmission term and variation in initial conditions and fit this model to observed data using Bayesian framework. Overall, the paper offers insight into how differences in initial conditions (i.e. introduction delays and initial susceptibility) can drive fine-scale spatio-temporal asynchrony in population dynamics.

## 2. Results

### 2.1. Asynchrony in pertussis epidemics associated with introduction delays

First, we begin by characterizing the spatio-temporal dynamics of the pertussis epidemic in Republic of Korea. The outbreak consisted of two waves that peaked around late July and December 2024 (figure 1*A*) and exhibited large spatial heterogeneity in timing (figure 1*B,C*) and magnitude (figure 1*D*). For example, both Seoul, the capital city located in the northwest region, and Busan, the second most populated city located at the southeast end, experienced two waves, whereas Daegu, the fourth most populated city located <100 km away from Busan, only experienced the second wave (figure 1*C*; see supplementary material, figure S1, for epidemic dynamics in other regions).

To further quantify spatial heterogeneity in pertussis waves across Republic of Korea, we calculate the centre of gravity (figure 1*E*), which represents the mean timing of cases [1]. Notably, we find a sharp contrast between early (orange) and late (light blue) waves between many neighbouring municipalities (figure 1*E*), suggesting spatial asynchrony in pertussis dynamics. To systematically evaluate this apparent asynchrony, we estimate the spatial synchrony function, which captures changes in correlation in epidemic dynamics as a function of distance [7]. The spatial synchrony function confirms a sharp reduction in cross-correlation with distance (figure 1*F*). For example, within 100 km of distance, the cross-correlation drops below 0.25. By comparison, pertussis epidemics in the USA maintained cross-correlations above 0.2 even at 1000 km [26], highlighting the fine-scale asynchrony in pertussis epidemics in Republic of Korea. Interestingly, the variation in the centre of gravity significantly correlates with the introduction timing (ρ=0.53; 95% CI: 0.44–0.62; figure 1*G*), suggesting that differences in introduction timing may have contributed to the apparent spatial asynchrony.

### 2.2. Synchrony in temporal changes in pertussis transmission

We then ask whether the observed epidemic asynchrony can be explained by differences in transmission patterns. To do so, we quantify the effective reproduction number R(t), which measures the average number of new infections caused by a single infected individual [27,28]. For this analysis, we focus on municipalities with more than 400 total cases reported because R(t) estimates can be unstable when the number of infections is low.

**Figure 2.**
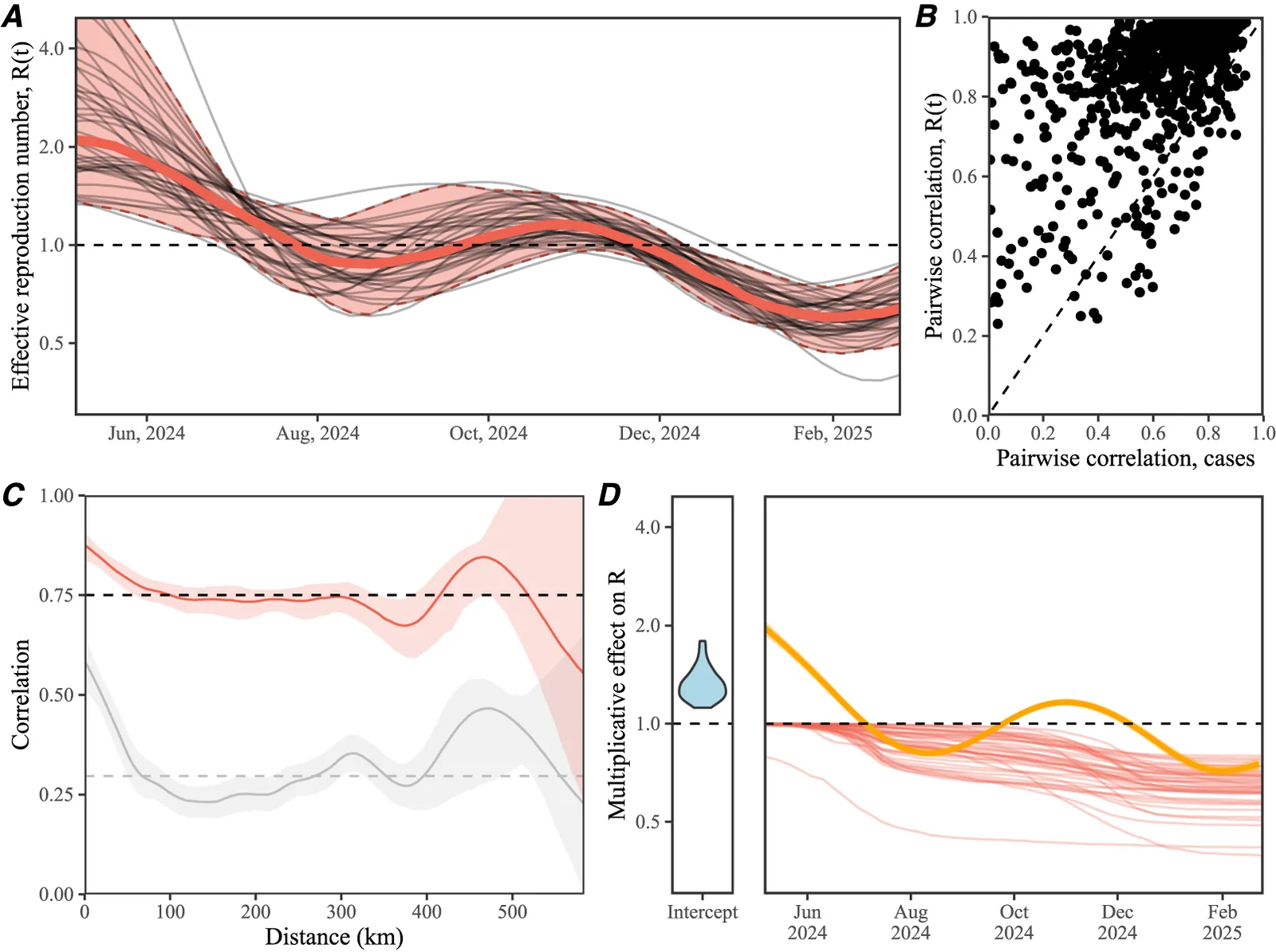
Spatial variation in the estimates of effective reproduction number. (*A*) Each black line represents the estimated effective reproduction number for a given municipality with more than 400 total cases. The red solid line and shaded regions represent the median and 95% quantiles of effective reproduction number estimates. (*B*) Relationship between correlation coefficient for R(t) and correlation coefficient for reported cases across all pairwise municipality combinations with more than 400 total cases. (*C*) Spatial synchrony in R(t) (red) versus logged cases (black) as a function of distance. The solid line and shaded regions represent the median estimate and the corresponding 95% confidence interval. The dashed horizontal line represents the mean correlation. (*D*) Estimated multiplicative effects on R(t) from the regression analysis. The blue shaded violin represents the distribution of intercept effect, where a separate intercept term is estimated for each municipality. The red lines represent the estimated susceptible depletion effect for each municipality. The orange line and shaded regions represent the estimated changes in transmission from a smooth term and the corresponding 95% confidence interval.

Notably, R(t) estimates appear to be largely homogeneous across different regions, despite the apparent heterogeneity in the observed epidemic dynamics (figure 2*A*). Comparisons of correlation coefficients in R(t) versus correlation coefficients in reported cases between all pairwise municipality combinations confirm this observation (figure 2*B*). In 788 out of 861 municipality combinations, correlation in R(t) is higher than correlation in cases. This is reflected in the spatial synchrony function for R(t) (figure 2*C*), where the mean cross-correlation for R(t) (ρ=0.75) is considerably higher than that for cases (ρ=0.30).

**Figure 3.**
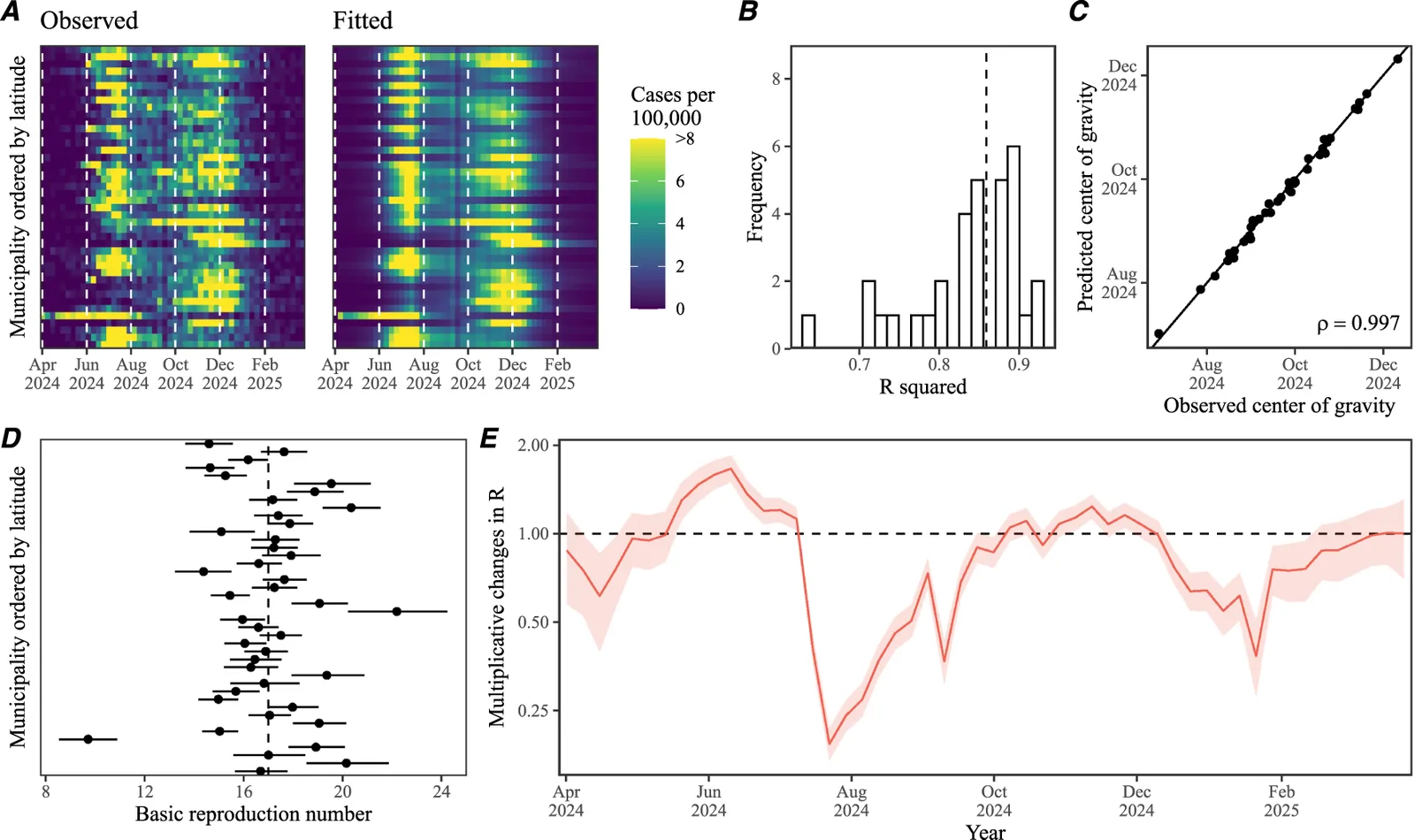
Fine-scale spatio-temporal variation in the pertussis epidemic can be explained with a simple susceptible–exposed–infected–removed (SEIR) model. (*A*) Comparisons between the observed and predicted epidemic dynamics across municipalities with more than 400 total cases, ordered by latitude. (*B*) The bar plot represents the distribution of R squared values for model fits. The vertical dashed line represents the median. (*C*) Relationship between the estimated and predicted centre of gravity. The solid line indicates the one-to-one relationship. (*D*) Estimated basic reproduction number $R_{0}$ across municipalities, ordered by latitude. Points and error bars represent posterior medians and corresponding 95% credible intervals. (*E*) Estimated time-varying pertussis transmission multiplier. The red line and shaded regions represent the posterior median and the corresponding 95% credible interval.

To better understand factors driving changes in R(t), we perform a regression analysis using a generalized additive model with three covariates [29–31]: (i) a municipality-level fixed effect allowing each municipality to have its own intercept, (ii) a cumulative-cases term capturing the effect of susceptible depletion, and (iii) a single smooth term shared across municipalities, which allows for temporal changes in pertussis transmission over time, such as seasonal forcing. First, the smooth term suggests a strong transmission reduction around August and February, which coincide with the timing of school breaks (figure 2*D*). Second, the model estimates a significant effect of susceptible depletion (figure 2*D*; p<0.001). Notably, this simple regression model explains 81% of variance in R(t), suggesting that the observed temporal fluctuations in transmission can be largely explained by school-related seasonality and the gradual depletion of susceptible individuals. Taken together, the analyses of centre of gravity and R(t) suggest that differences in introduction timing and susceptible host dynamics are likely main drivers for spatial asynchrony in pertussis epidemics in Republic of Korea, rather than variation in seasonal transmission patterns. This analysis provides a key motivation for developing and parametrizing a mechanistic model to explain the apparent asynchrony in pertussis epidemic dynamics.

### 2.3. Mathematical modelling of fine-scale spatio-temporal pertussis epidemic

To test whether variation in introduction timing and susceptible host dynamics can explain the observed epidemic patterns, we extend a standard susceptible–exposed–infected–removed (SEIR) model [32] to jointly estimate a single, time-varying transmission term, which is shared across all regions. In particular, the transmission rate $\beta_{r}(t)$ in region r is modelled as follows:

(2.1) $$\beta_{r}(t)=\beta_{r}(0)\delta (t),$$

where $\beta_{r}(0)$ represents the baseline transmission rate, which accounts for variation in baseline transmission levels and is estimated separately for each region r. Then, δ(t) represents a time-varying transmission term, which is shared across all regions and captures a multiplicative change in transmission over time. We also allow the initial susceptible fraction S(0) to be shared across all regions to capture the average level of susceptibility. Since S(0) is shared across regions, the estimated regional variation in $\beta_{r}(0)$ should be interpreted as variation in baseline transmission potential, rather than contact variation alone. In particular, any unmodelled spatial variation in susceptibility, age-specific immunity or contact patterns may be partially absorbed into the estimate of $\beta_{r}(0)$. Initial infected fraction $I_{r}(0)$ and reporting rate $\rho_{r}$ are allowed to vary across regions, where variation in I(0) effectively captures differences in introduction timing. For this analysis, we also focus on municipalities with more than 400 total cases. The model is fitted using the Bayesian inference software rstan [33].

This simple transmission model that assumes shared transmission rate can capture fine-scale spatio-temporal variation in the pertussis outbreak in Republic of Korea (figure 3*A*) with a median R squared value of 0.86 (95% quantile: 0.71–0.92; figure 3*B*). Furthermore, we find a near-perfect correlation between the predicted and observed centre of gravity (figure 3*C*). These results suggest that the model assuming shared time-varying transmission can sufficiently explain the fine-scale spatial asynchrony observed during the pertussis outbreak in Republic of Korea. So, how do transmission levels vary across regions and transmission change across time?

We estimate a considerable variation in $R_{0}$ across regions (figure 3*D*). The median estimates for region-level $R_{0}$ vary from 9.7 to 22.1 with an average of 17, consistent with previous estimates [20,34,35]. The estimated initial susceptible fraction is 0.15 (95% CI: 0.14–0.16), which corresponds to an initial effective reproduction number of 2.6 when combined with the average $R_{0}$ estimate—this is consistent with the initial R(t) estimate. Finally, we also estimate a large reduction in transmission around late July 2024 and late January 2025 (figure 3*E*). The estimated timing of transmission reduction is also consistent with results from the R(t) analysis and coincides with the timing of school holidays in Republic of Korea. Parameter estimates for the initial infected fraction I(0) and reporting rates also reveal large variation across regions (supplementary material, figure S2). While estimates of $R_{0}$ or I(0) do not correlate with logged population sizes, reporting rate negatively correlates with population size (supplementary material, figure S3). By contrast, population density shows a weak, positive correlation with estimates of $R_{0}$ (supplementary material, figure S3; ρ=0.38; 95% CI: 0.08–0.61; p=0.01 on the original scale; ρ=0.30; 95% CI: 0.00–0.55; p=0.053 after log transformation). We did not find significant correlations between estimated parameters and school-aged population fraction (supplementary material, figure S3).

We validate the model by taking the estimated transmission term δ(t) and fitting the remaining parameters (i.e. initial infected, initial susceptible, basic reproduction number and reporting probability) to data from the remaining 210 municipalities with fewer than 400 total cases (supplementary material, figure S4). While model predictions appear consistent with the observed patterns (supplementary material, figure S4A), we estimate a lower value for the median R squared: 0.59 (95% quantile: 0.01–0.85; supplementary material, figure S4B). Nonetheless, this analysis suggests that the majority of asynchrony in pertussis epidemics can be explained via differences in introduction timing and transmission variation.

As an alternative model, we also consider the possibility that the spatio-temporal asynchrony may be driven by variation in susceptibility. To do so, we assume a fixed basic reproduction number, $R_{0}=17$, and estimate a separate initial susceptible fraction $S_{r}(0)$ for each region and a shared time-varying transmission term δ(t). This alternative model provides nearly identical fits to data compared to the original model (supplementary material, figures S5 and S6) and similar levels of out-of-sample validation (supplementary material, figure S7). While we do not find significant correlation between past vaccine coverage and the estimated levels of susceptibility (supplementary material, figure S8), these observations do not provide evidence against spatial variation in susceptibility because the 2015 vaccine coverage data provide only a crude proxy for population-level susceptibility during the 2024–2025 outbreak and do not capture potential differences in waning immunity, booster uptake, prior infection history or age-specific susceptibility.

A formal model comparison based on the widely applicable information criterion (WAIC) and Pareto-smoothed importance sampling leave-one-out cross-validation (PSIS-LOO) favoured the model with spatially varying $R_{0}$, rather than the alternative model with spatially varying S(0) (ΔWAIC = 86.2; ΔELPD = 33.0, SE = 12.7). However, both comparisons should be interpreted with care because both WAIC and PSIS-LOO diagnostics indicated instability in model comparisons. Thus, these results do not provide evidence against spatial variation in susceptibility. Instead, they suggest that spatial variation in baseline transmission potential and spatial variation in susceptibility provide alternative explanations that are difficult to distinguish from case data alone; in fact, the estimated municipality-level $R_{0}$ and S(0) from two separate models are almost perfectly correlated (ρ=0.996), indicating parameter unidentifiability (supplementary material, figure S9). We therefore use the model with spatial variation in $R_{0}$ as the primary model for subsequent analyses, while acknowledging that spatial heterogeneity in susceptibility remains an equally plausible mechanism that contributes to the observed asynchrony.

**Figure 4.**
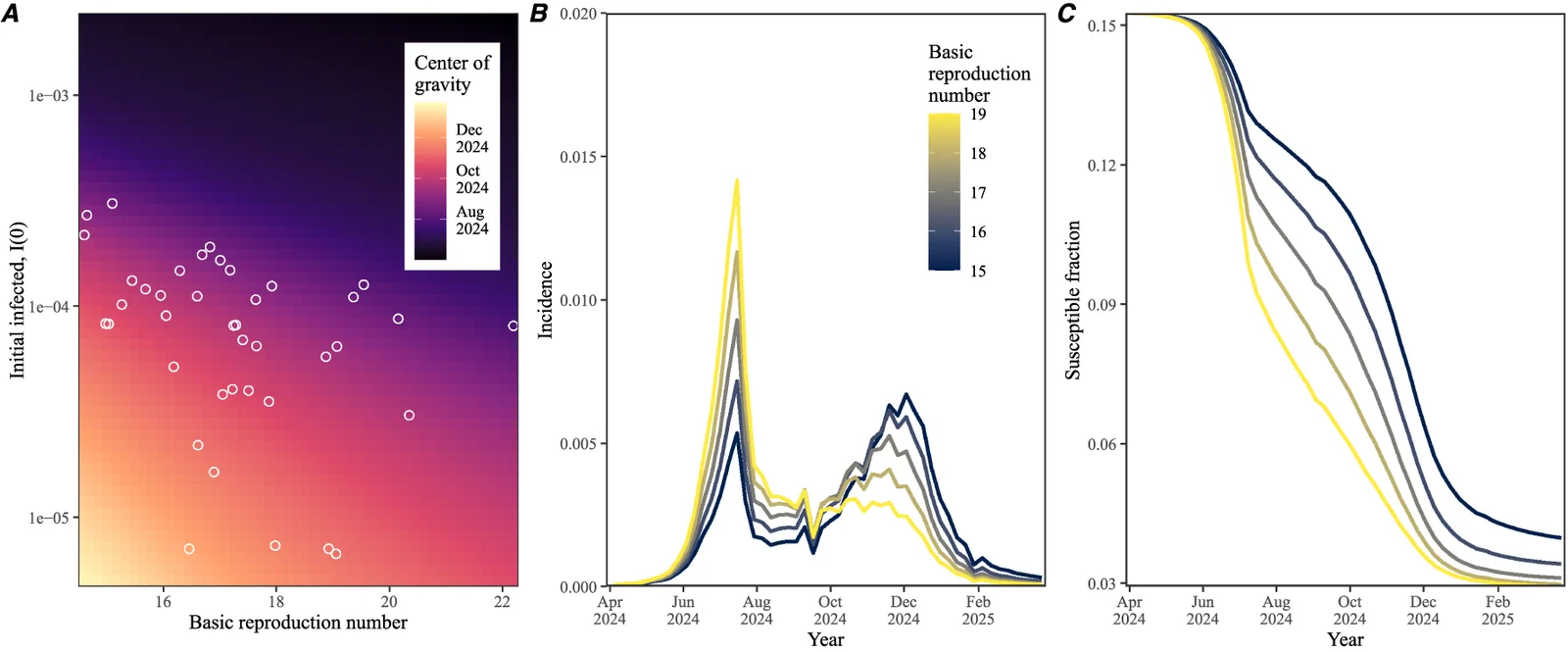
Impact of delayed introduction, transmission variation and susceptible host dynamics on epidemic waves. (*A*) Relationship between the basic reproduction number $R_{0}$ and infected I(0) fraction with centre of gravity. Points represent the estimates for each municipality. (*B*) Impact of basic reproduction number on pertussis epidemic waves. (*C*) Impact of basic reproduction number on pertussis susceptible host dynamics. Colours in (*B*,*C*) represent values of basic reproduction number.

So, how does variation in introduction timing and transmission potential generate heterogeneity in epidemic waves? We address this question by simulating the model between April 2024 and April 2025 across plausible ranges of $R_{0}$ and I(0). First, increasing I(0) leads to an earlier epidemic because it permits earlier introduction of the infection to the population (figure 4*A*). This is consistent with the earlier observations (figure 1*F*). Similarly, increasing $R_{0}$ also results in an earlier epidemic. This is because high levels of transmission allow for a large first wave (figure 4*B*), which prevents the second wave through the overcompensatory susceptible depletion (figure 4*C*). By contrast, when $R_{0}$ is low, there will be enough susceptible hosts for the population to experience the second wave (figure 4*C*). These results are consistent with an earlier finding, which showed that transmission variation can explain a sudden shift in RSV seasonality from a later wave to an earlier wave [36].

### 2.4. Relative roles of delayed introduction, transmission variation and demographic stochasticity in driving spatial asynchrony

Finally, previous studies indicated that pertussis transmission can be particularly sensitive to demographic stochasticity compared to other childhood infections, such as measles. So far, we have used deterministic models to tease apart how delayed introduction and transmission variation can generate heterogeneity in epidemic waves, but this does not rule out the possibility that demographic stochasticity may have contributed to spatial asynchrony in pertussis outbreaks.

To evaluate the relative roles of demographic stochasticity, introduction timing and transmission variation, we simulate a stochastic SEIR model for each municipality under four different assumptions and compare the mean of all pairwise correlation coefficients in simulated reported cases. First, we considered a baseline scenario in which all municipalities had identical basic reproduction number, $R_{0}$, and initial infected fraction, I(0), where I(0) serves as a proxy for introduction timing. This scenario tests whether demographic stochasticity alone can generate the observed spatial asynchrony. Second, we allowed I(0) to vary across municipalities by sampling with replacement from the median posterior estimates, while holding $R_{0}$ fixed. Third, we allowed $R_{0}$ to vary across municipalities by sampling with replacement from the median posterior estimates, while holding I(0) fixed. Finally, we allowed both $R_{0}$ and I(0) to vary across municipalities by resampling both parameters independently. This resampling approach preserves the empirical variation in baseline transmission and introduction timing estimated from the deterministic model.

**Figure 5.**
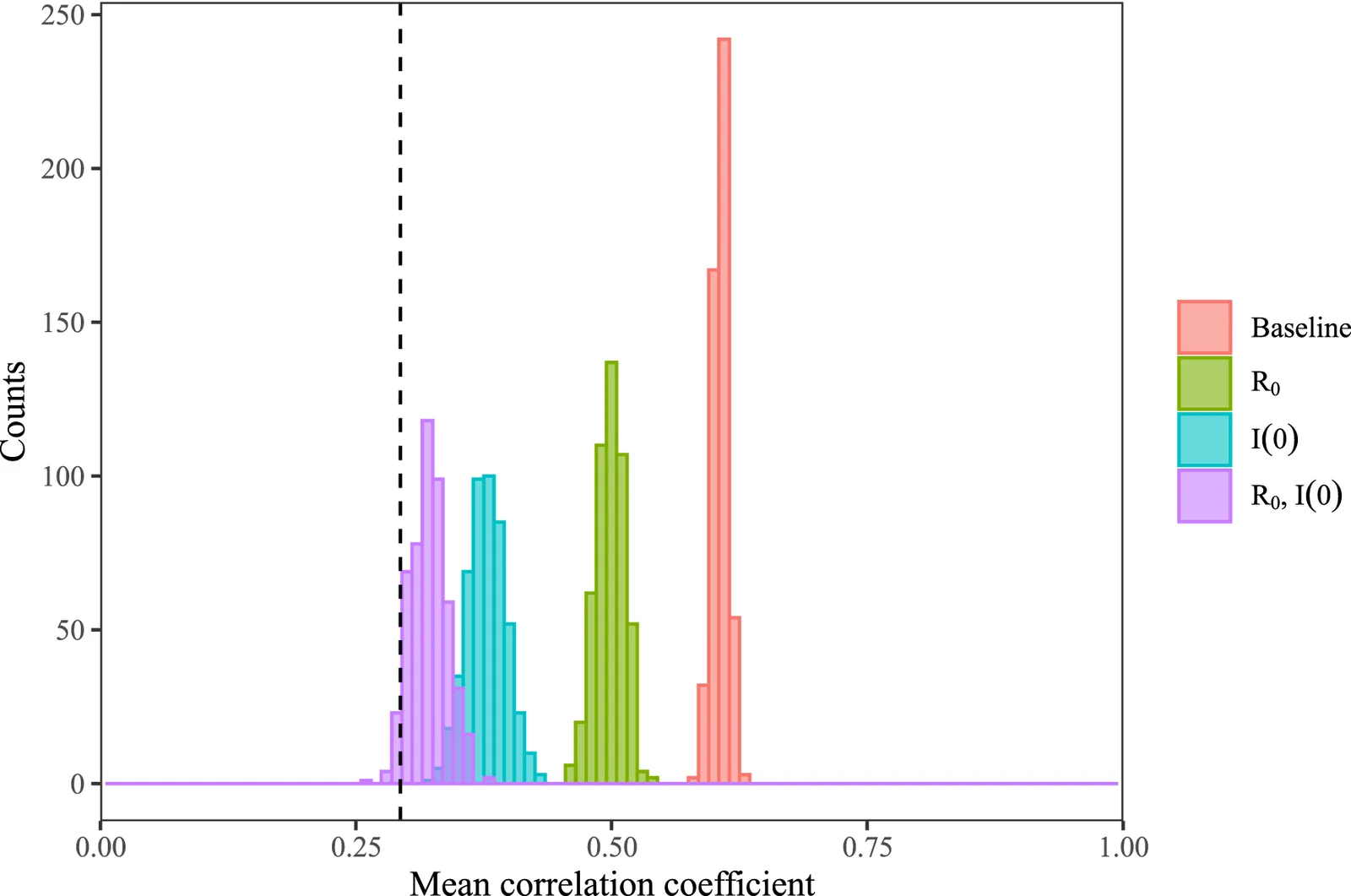
Effects of demographic stochasticity, introduction timing and transmission variation on spatial synchrony. Coloured histograms represent the simulated distributions of mean pairwise correlation coefficients across all municipalities under four different assumptions: (i) identical $R_{0}$ and I(0) across all regions (baseline); (ii) identical I(0), but municipality-specific $R_{0}$ ($R_{0}$); (iii) identical $R_{0}$, but municipality-specific I(0) (I(0)); (iv) municipality-specific $R_{0}$ and I(0) ($R_{0}$, I(0)). Municipality-specific $R_{0}$ and I(0) are obtained by resampling with replacement from a distribution of posterior median estimates. The dashed vertical line represents the observed mean value of all pairwise correlation coefficients.

Overall, we find that demographic stochasticity alone cannot reproduce the observed levels of spatial asynchrony (figure 5). Allowing for variation in $R_{0}$ and I(0) reduces the degree of spatial synchrony but the simulated levels of spatial synchrony, captured by the distributions of mean pairwise correlation coefficients, are still higher than the observed levels. Instead, the simulations support the main conclusion from the deterministic model: spatial variation in introduction timing I(0), together with variation in baseline transmission $R_{0}$, can generate spatial asynchrony even when all municipalities share the same time-varying transmission pattern δ(t).

## 3. Discussion

Understanding mechanisms that drive spatio-temporal variation in epidemic dynamics is a major aim for infectious disease dynamics and control [1–4,7–9]. The large pertussis outbreak in Republic of Korea that occurred between 2024 and 2025 presents a unique perspective on fine-scale spatio-temporal heterogeneity in epidemic dynamics: some municipalities experienced two epidemic waves while other places experienced only the first or second without clear patterns of synchrony. We propose that this variation can be explained by differences in introduction delays, transmission variation and susceptible host dynamics, where high levels of transmission can cause a large first wave and prevent the second wave through susceptible depletion. Incorporating this idea into a simple transmission model by allowing for a joint estimation of a shared transmission term confirms this hypothesis. Together, these findings shed light on mechanisms that can generate spatial asynchrony in epidemic dynamics, offering a general framework for understanding the spatio-temporal dynamics of re-emerging infections.

Although the main model used for parameter estimation is deterministic, the resulting inference still hints at the role of stochasticity in driving the observed spatial asynchrony. Here, we distinguish between two phases of stochasticity that can affect the observed pertussis waves. The first is stochasticity during initial establishment, which affects introduction timing and subsequent epidemic take-off [7,37,38]. In reality, this process is largely stochastic, where introductions from other municipalities and local extinction determine when sustained local transmission begins. The deterministic model provides a simple way to approximate this process, where a lower initial infected fraction corresponds to a later introduction and slower epidemic take-off. As shown in figure 5, this form of stochasticity is likely important for explaining the observed spatial asynchrony.

The second is demographic stochasticity in transmission after the epidemic takes off [39–41]. This alone appears insufficient to explain the observed dynamics, but it can still contribute to spatial heterogeneity in epidemic trajectories. Therefore, we tentatively conclude that the observed spatial asynchrony is primarily driven by a combination of stochastic variation in introduction timing and deterministic variation in baseline transmission.

Even though the model presented in this paper explains the observed dynamics reasonably well, there are several limitations to this work. For example, while population density was weakly associated with the estimated transmission potential, we were unable to fully identify the mechanisms that explain this variation. Other municipal-level covariates, such as age structure, school enrollment, urban/rural classification, mobility and local contact patterns, may also contribute to spatial variation in transmission. The school-aged population fraction, a simple proxy for age structure and school-related mixing, was not significantly associated with the estimated parameters. Other municipal-level covariates, such as urban/rural classification, mobility and local contact patterns, may also contribute to spatial variation in transmission.

As an alternative model, we also considered the possibility that spatial asynchrony can be explained by susceptible variation given the role of waning immunity in driving pertussis re-emergence [20,42,43]. Although we did not find a clear correlation between past vaccine coverage and estimated variation in susceptible fraction, this analysis does not rule out spatial variation in susceptibility. Detailed serological data may allow us to test different model formulations and further quantify potential variation in population-level susceptibility [44–46]. Such analyses will require careful consideration of the distinction between seropositivity and protection against infection, as well as the effect of natural immune boosting on serological measurements [47].

Resulting parameter estimates must be interpreted with care as the model relies on several simplifying assumptions. For example, the main model assumed the same values of initial susceptible fraction across all municipalities and tried to estimate separate basic reproduction number $R_{0}$. Therefore, any regional variation in S(0), driven by differences in vaccine coverage and waning, would be captured in the estimate of $R_{0}$. We also did not consider an age structure in the model, but age-structured contact patterns play an important role in shaping pertussis transmission [32]. For example, Cho *et al.* [48] recently suggested transmission among elderly may have contributed to the persistence of pertussis in Republic of Korea. Differences in age structure across regions may also contribute to variation in transmission rates and population-level immunity [49], but detailed age-structured data would be needed to address these challenges. Finally, we modelled time-varying transmission phenomenologically using a single δ(t) term, which revealed that the timing of transmission reduction coincided with school holidays in Republic of Korea, but other factors may also contribute to temporal changes in transmission. In particular, acellular pertussis vaccine immunity can wane at rates of 2–10% yr^–1^ [50–52], which in turn may affect epidemic dynamics over the course of an outbreak. Such effects may also be partially captured by the time-varying transmission term δ(t), and therefore δ(t) must be interpreted with care. Despite these limitations, this work provides a quantitative foundation and empirical evidence for understanding epidemic asynchrony.

The continued emergence of new pathogens and the re-emergence of vaccine-preventable infections, such as pertussis, highlight the importance of identifying hotspots of future outbreaks and developing targeted intervention strategies [45,53,54]. The apparent asynchrony in pertussis epidemics in Republic of Korea, driven by differences in introduction timing and transmission potential, underlines the importance of detailed pathogen surveillance for early detection and strategic allocation of prevention and control resources. Immunological surveillance platforms will be particularly useful for evaluating potential risk of pathogen re-emergence in the future [44–46]. A detailed understanding of spatial variation in transmission patterns and population-level immunity will be crucial for predicting spatio-temporal dynamics of future outbreaks.

## 4. Material and methods

### 4.1. Data

The weekly surveillance data on pertussis cases across 252 municipalities (first week of 2024 to the 44th week of 2025) were obtained from a publicly available website, the Infectious Disease Portal, by the Korea Disease Control and Prevention Agency [55]. Population size data as of September 2025 were obtained from a publicly available website by the Ministry of the Interior and Safety [56]. Municipality-level vaccine coverage data among 3-year-old children as of 2015 were obtained from a publicly available website maintained by the Korea Disease Control and Prevention Agency [57]. Because the Korean DTaP schedule recommends doses at two, four, six and 15–18 months, coverage among 3-year-old children was interpreted as coverage for the fourth DTaP dose. The 2015 data were used because they were the oldest vaccine coverage data publicly available at the municipality level. Longitude and latitude data for each municipality were obtained from a publicly available GitHub repository [58]. Municipality-level data on school-aged population fraction (i.e. the fraction of population aged 5–19 years) were obtained from the Korean Statistical Information Service website [59]. Finally, shape files used for constructing maps were obtained from a publicly available website [60].

### 4.2. Centre of gravity

We quantified the centre of gravity to characterize variation in the mean timing of the epidemic. Typically, the centre of gravity is calculated based on annual incidence for recurrent epidemics. Since the paper focuses on analysing a single outbreak, we used the entire time series of reported cases $C_{t}$ to compute the centre of gravity for each municipality:

(4.1) $$\text{centre of gravity}=\frac{\sum_{t=1}^{t_{\text{max}}}C_{t}\times t}{\sum_{t=1}^{t_{\text{max}}}C_{t}}.$$

We then calculated the correlation coefficient between the centre of gravity and the timing of introduction, defined as the first week when the number of reported cases is greater than 1 in 100 000.

### 4.3. Spatial synchrony of reported cases

We characterized the spatial synchrony of the pertussis epidemic, which captures changes in pairwise correlation as a function of distance [7]. We used logged values of reported cases + 1 between May 2024 and March 2025, excluding municipalities that had no reported cases (n=250). Spatial synchrony was calculated using the ncf package in R [61]. We used 1000 bootstraps to generate the median estimate and the corresponding 95% confidence intervals.

### 4.4. Effective reproduction number

We calculated the effective reproduction number R(t) to characterize changes in pertussis transmission over time. First, we took the case time series $C_{t}$ and fitted a generalized additive model assuming a negative binomial error to smooth the time series [30]. Then, we estimated R(t) using the method of Cori *et al.* [28]:

(4.2) $$R(t)=\frac{i(t)}{\sum_{k=1}^{n}i(t-k)g(k)},$$

where i(t) is the smoothed incidence and n is the maximum length of generation interval in weeks (assumed to be five weeks). The generation-interval distribution g(k) was assumed to follow a gamma distribution with a mean of 9.47 days and a standard deviation of 6.22 days based on the estimated serial-interval distribution during this outbreak [62]. The reproduction number estimates can be unrealistically high when the number of infections is close to zero. Therefore, we only used estimates between May 2024 and March 2025 throughout the paper.

First, we compared correlation coefficients between R(t) estimates and those between logged cases across all pairwise municipality combinations (figure 2*B*). For this analysis, we only used data from municipalities with more than 400 total cases. Then, we compared the spatial synchrony for R(t) estimates and synchrony for logged cases (figure 2*C*). For this analysis, we used R(t) estimates from all municipalities without any exclusion.

Finally, we fitted a generalized additive model to logged values of R(t) to quantify the effect of susceptible depletion and temporal variation in intrinsic transmission [29–31]:

(4.3) $$\log\left(R_{t,m}\right)=\alpha_{m}+\beta d_{t,m}+s(t),$$

where $R_{t,m}$ represents the R(t) estimate at time t in municipality m, $\alpha_{m}$ represents the municipality-specific intercept term, β represents the effect of susceptible depletion, $d_{t,m}$ represents the cumulative cases at time t in municipality m and s(t) represents a non-parametric smooth function of time, capturing temporal variation in transmission. The smooth term s(t) was estimated within the generalized additive model, and its estimation did not require a prespecified parametric form. For this analysis, we only used R(t) estimates from municipalities with more than 400 total cases.

Although the serial-interval distribution with a mean of 9.47 days and a standard deviation of 6.22 days were estimated from the same pertussis outbreak in Republic of Korea, its mean is considerably shorter than that of previous estimates, which can be nearly three weeks long [63]. As discussed by Akhmetzhanov *et al.* [64], these differences may reflect contact tracing and isolation efforts: using the same serial-interval data from Republic of Korea, Akhmetzhanov *et al.* [64] estimated the counterfactual unmitigated serial-interval distribution to have a mean of 14.7 days. As a sensitivity analysis, we repeated the analysis by doubling the mean and standard deviation of the serial-interval distribution (supplementary material, figure S10). The overall conclusions were robust to this change.

### 4.5. Transmission model

Finally, we fitted a simple transmission model using Bayesian inference to test whether variation in introduction timing and transmission variation alone can explain the observed epidemic patterns. Specifically, we extended the SEIR model, which is commonly used for pertussis transmission [32], to allow for a joint estimation of a single, time-varying transmission term that is shared across all regions and a separate estimation of initial infected fraction, basic reproduction number and reporting rates. To do so, we first discretized the SEIR model following the approach of He *et al.* [65]:

(4.4)βm(t)=R0,m(1−exp⁡(−γΔt))δ(t),(4.5)FOIm(t)=βm(t)Im(t−Δt)Nm,(4.6)ΔSm(t)=[1−exp⁡(−FOIm(t)Δt)]Sm(t−Δt),(4.7)ΔEm(t)=[1−exp⁡(−σΔt)]Em(t−Δt),(4.8)ΔIm(t)=[1−exp⁡(−γΔt)]Im(t−Δt),(4.9)Sm(t)=Sm(t−Δt)−ΔSm(t),(4.10)Em(t)=Em(t−Δt)−ΔEm(t)+ΔSm(t)(4.11)andIm(t)=Im(t−Δt)−ΔIm(t)+ΔEm(t),

where $S_{m}(t)$, $E_{m}(t)$ and $I_{m}(t)$ represent the number of susceptible, exposed and infectious individuals in municipality m at time t, respectively; $N_{m}$ represents the population size of municipality m; Δt represents the simulation time step, which was assumed to be one week; $\beta_{m}(t)$ represents the shared time-varying transmission rate; $R_{0}$ represents the shared basic reproduction number [66]; δ(t) represents the normalized time-varying transmission rate; σ represents the rate at which individuals develop infectiousness, which is assumed to be σ=−log⁡(1−7/8) such that the mean latent period is 8 days; γ represents the rate at which individuals recover, which is assumed to be γ=−log⁡(1−7/15) such that the mean infectious period is 15 days.

Here, we modelled changes in transmission using the δ(t) term, which is given a normal prior around 1:

(4.12) δ(t)∼normal(1,0.2).

To allow for smooth variation in transmission, we also incorporated a random walk prior:

(4.13)δ(t)∼normal(δ(t−Δt),σδ)(4.14)andσδ∼half-normal(0,0.1),

where a half-normal prior on $\sigma_{\delta }$ constrains the smoothness of δ(t). We also estimated a municipality-specific basic reproduction number:

(4.15) $$\begin{array}{r} R_{0,m}∼\mathrm{normal}(17,2). \end{array}$$

The municipality-specific observation process is modelled based on a Poisson distribution:

(4.16)casest,m∼poisson(ρmΔSm(t))(4.17)andρm∼uniform(0,1),

where ${\mathrm{cases}}_{t,m}$ represents the reported cases at time t in municipality m, $\rho_{m}$ represents the municipality-specific reporting rate and $\Delta S_{m}(t)$ represents the expected number of new infections between time t−Δt and t.

Finally, we estimated a municipality-specific initial infected fraction $i_{m}(0)$ and a shared initial susceptible fraction s(0) by imposing the following priors:

(4.18)s(0)∼uniform(0,1)(4.19)andim(0)∼half-normal(0,0.001).

Then, the initial conditions for the model are specified as follows:

(4.20)S(0)=Nms(0),(4.21)Em(0)=Nmim(0)(4.22)andIm(0)=Nmim(0).

We simultaneously fitted this model to time-series data between May 2024 and March 2025 from all municipalities with more than 400 total cases (a total of 42 municipalities) using the Bayesian inference software rstan with four chains, each with 2000 iterations [33]. Convergence was assessed by ensuring low R-hat (<1.01), high effective sample size (>800), no divergent transitions, and no iterations that exceeded the maximum tree depth.

To assess the model fit, we computed R squared values for each municipality. This is done by calculating the squared value of the correlation coefficient between logged values of the observed cases + 1 and a posterior median of the logged predictions $\rho_{m}\Delta S_{m}(t)$.

Finally, we evaluated the impact of initial conditions on pertussis epidemic dynamics by varying $R_{0}$ between 14.2 and 22.2 and i(0) between $5\times 10^{-6}$ and $2.3\times 10^{-3}$. For each simulation, we quantified the centre of gravity.

As an alternative model, we also considered a model assuming identical $R_{0}$ across municipalities and estimated a municipality-specific initial susceptible fraction $s_{m}(0)$. The same prior distributions were used for this analysis.

Since Poisson error structure can result in overly narrow confidence intervals and biased parameter estimates [67,68], we also considered a model assuming negative binomial observation errors:

(4.23)casest,m∼negative-binomial(ρmΔSm(t),k)(4.24)andk∼half-normal(0,10),

where k represents the shared overdispersion parameter. The same prior distributions were used for all other parameters. This gave slightly wider credible intervals for both the basic reproduction number $R_{0}$ and time-varying transmission term δ(t) with overall similar model fits, providing robustness to the analysis (supplementary material, figure S11).

### 4.6. Transmission model validation

We validated the model by fitting the model to the remaining municipalities (i.e. those with less than 400 total cases). In doing so, we assumed that changes in transmission rate δ(t) were known and only estimated the initial conditions, s(0) and $i_{m}(0)$, basic reproduction number $R_{0,m}$ and the reporting rate $\rho_{m}$. For this validation, we used the optimization function in rstan [33] rather than performing a full Bayesian inference. Then, we computed R squared values for each municipality in the same way as before.

### 4.7. Relationship between estimated initial susceptible and vaccine coverage

We assessed the relationship between estimated initial susceptible from the alternative model and vaccine coverage using linear regression. Specifically, assuming that the waning of vaccinal immunity is the main cause for pertussis re-emergence, we compared DTaP vaccine coverage for 3-year-old children as of 2015 and the estimated initial susceptible fraction in each municipality and performed a linear regression. We used vaccine coverage as of 2015 because this was the oldest data that were publicly available. We considered using estimates from municipalities with >400 cases, since the parameter estimates are expected to be more reliable, but were unable to find any clear signature between vaccine coverage and estimated s(0).

### 4.8. Stochastic simulations

We used a stochastic version of the discrete-time SEIR transmission model to quantify the relative roles of demographic stochasticity, introduction timing and transmission variation in driving spatial asynchrony in pertussis epidemics. To do so, we considered the following four scenarios: (i) all municipalities have identical basic reproduction number, $R_{0}$, and initial infected fraction, I(0), where I(0) serves as a proxy for introduction timing; (ii) I(0) varies across municipalities, but $R_{0}$ remains fixed; (iii) $R_{0}$ varies across municipalities, but I(0) remains fixed; (iv) both $R_{0}$ and I(0) vary across municipalities. When a parameter was fixed, we used the median of the corresponding posterior median estimates. When a parameter was allowed to vary, municipality-level values were sampled with replacement from the posterior median estimates. For all simulations, we used median estimates for shared time-varying transmission term δ(t) and for initial susceptible fraction S(0) from the fitted transmission model.

For each scenario, we conducted 500 simulations. In each simulation, we simulated stochastic epidemic trajectories for all municipalities using their observed population sizes. Reported cases were then simulated from the expected incidence using the estimated median reporting rate and Poisson observation error. For each simulated dataset, we calculated pairwise correlations in logged reported cases, log⁡(C+1), between all municipalities and summarized spatial synchrony by taking the mean of all pairwise correlation coefficients. We then compared the resulting distributions of mean correlation coefficients across scenarios with the observed mean correlation coefficient. Model equations are provided in S1 in the supplementary material.

## Supplementary Material



## Data Availability

All data and code used in this paper are available at http://github.com/parklab-snu/asynchrony-pertussis and [69]. Supplementary material is available online [70].
