# Supplementary material for: Delayed introduction, transmission variation and susceptible dynamics explain spatial asynchrony during Republic of Korea's large pertussis outbreak
Source: J R Soc Interface. 2026 Oct 21;23(243):20260205. doi: 10.1098/rsif.2026.0205 (PMC13682875; doi:10.1098/rsif.2026.0205)
Supplement: Supplementary file 1 [file rsif.2026.0205.zip › rsif.2026.0205/rsif20260205_si_001.pdf]

Supplementary Materials

Delayed introduction, contact variation, and susceptible dynamics explain spatial asynchrony during Korea's large pertussis outbreak

Sang Woo Park[1,*]

1 School of Biological Sciences, Seoul National University, Seoul, Korea

*Corresponding author: sangwoopark@snu.ac.kr

Supplementary Text

S1 Stochastic simulation

A stochastic version of the SEIR model is implemented based on the Euler-multinomial scheme [1]:

$$\beta_m(t) = \mathcal{R}_{0,m}(1 - \exp(-\gamma \Delta t))\delta(t) \tag{1}$$

$$\text{FOI}_m(t) = \frac{\beta_m(t) I_m(t - \Delta t)}{N_m} \tag{2}$$

$$\Delta S_m(t) \sim \text{Binomial}\left(S_m(t - \Delta t), 1 - \exp(-\text{FOI}_m(t)\Delta t)\right) \tag{3}$$

$$\Delta E_m(t) \sim \text{Binomial}\left(E_m(t - \Delta t), 1 - \exp(-\sigma \Delta t)\right) \tag{4}$$

$$\Delta I_m(t) \sim \text{Binomial}\left(I_m(t - \Delta t), 1 - \exp(-\gamma \Delta t)\right) \tag{5}$$

$$S_m(t) = S_m(t - \Delta t) - \Delta S_m(t) \tag{6}$$

$$E_m(t) = E_m(t - \Delta t) - \Delta E_m(t) + \Delta S_m(t) \tag{7}$$

$$I_m(t) = I_m(t - \Delta t) - \Delta I_m(t) + \Delta E_m(t) \tag{8}$$

The observation model is assumed to follow the Poisson distribution:

$$\text{cases}_{t,m} \sim \text{Poisson}(\rho_m \Delta S_m(t)). \tag{9}$$

All parameters are defined as in the deterministic model.

Supplementary Figure

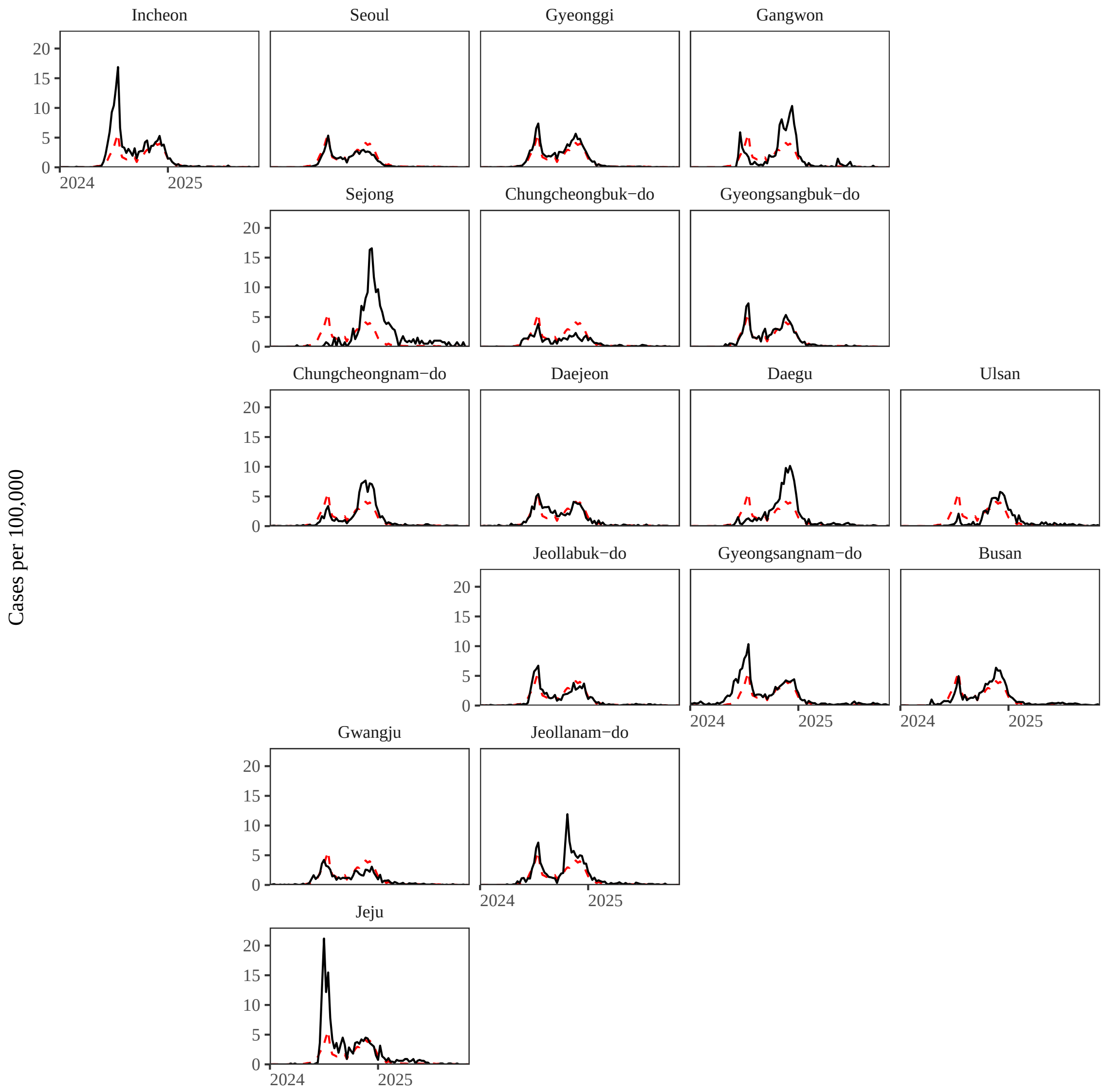

Figure S1: **Spatiotempordal dynamics of pertussis outbreak in Korea across first-level administrative divisions, 2024—2025.** Time series are arranged based on their approximate locations.

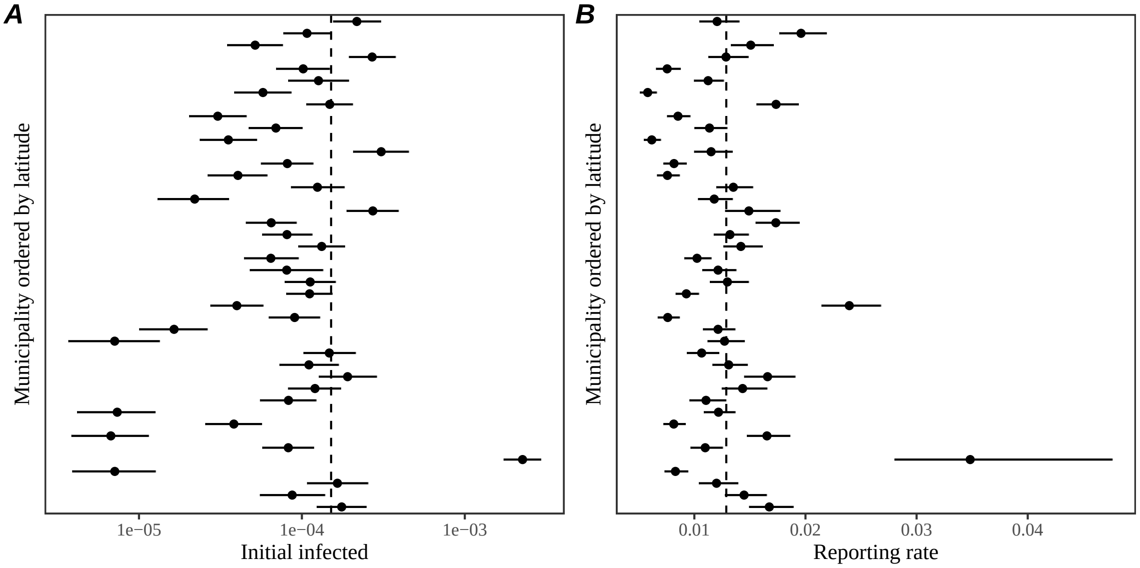

Figure S2: **Estimated initial infected fraction (A) and reporting rates (B) using time series data from municipalities with less than 400 total cases.** Points represent posterior medians. Error bars represent 95% credible intervals.

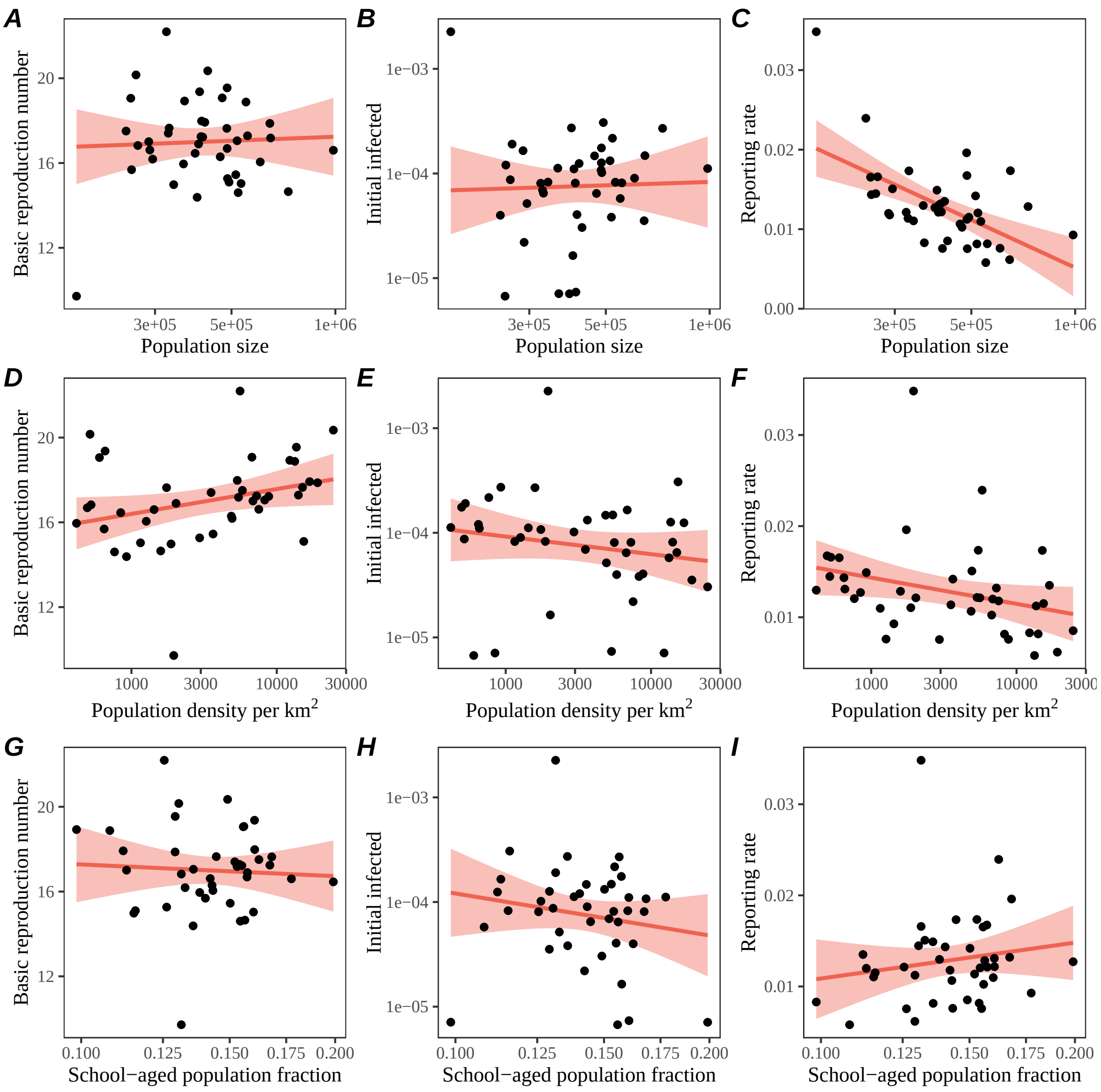

Figure S3: **Relationship between population size, density, school-aged population fraction and parameter estimates for the basic reproduction number (A,D,G), initial infected fraction (B,E,H), and reporting rate (C,F,I).** Points represent posterior medians. Error bars represent 95% credible intervals. Red lines and shaded regions represent the linear regression fit and associated 95% confidence intervals.

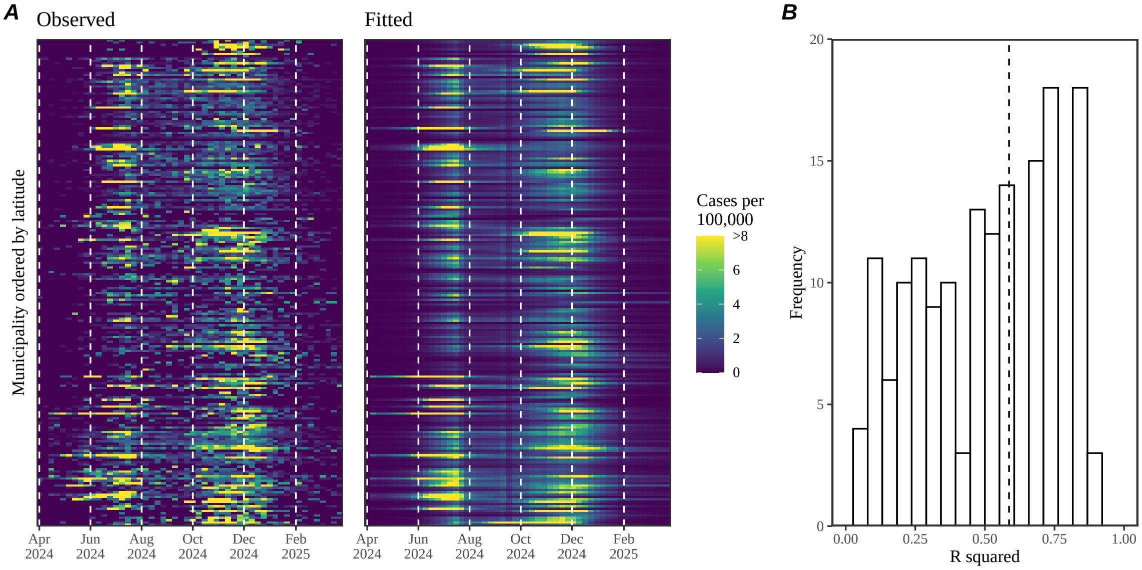

Figure S4: **Validation of the transmission model using time series data from municipalities with less than 400 total cases.** (A) Comparisons between the observed and predicted epidemic dynamics across municipalities with less than 400 total cases, ordered by latitude. (B) The bar plot represents the distribution of R squared values for model fits. The vertical dashed line represents the median. Initial infected, initial susceptible, basic reproduction number, and reporting probability were estimated for each region while assuming a known transmission variation term $\delta(t)$.

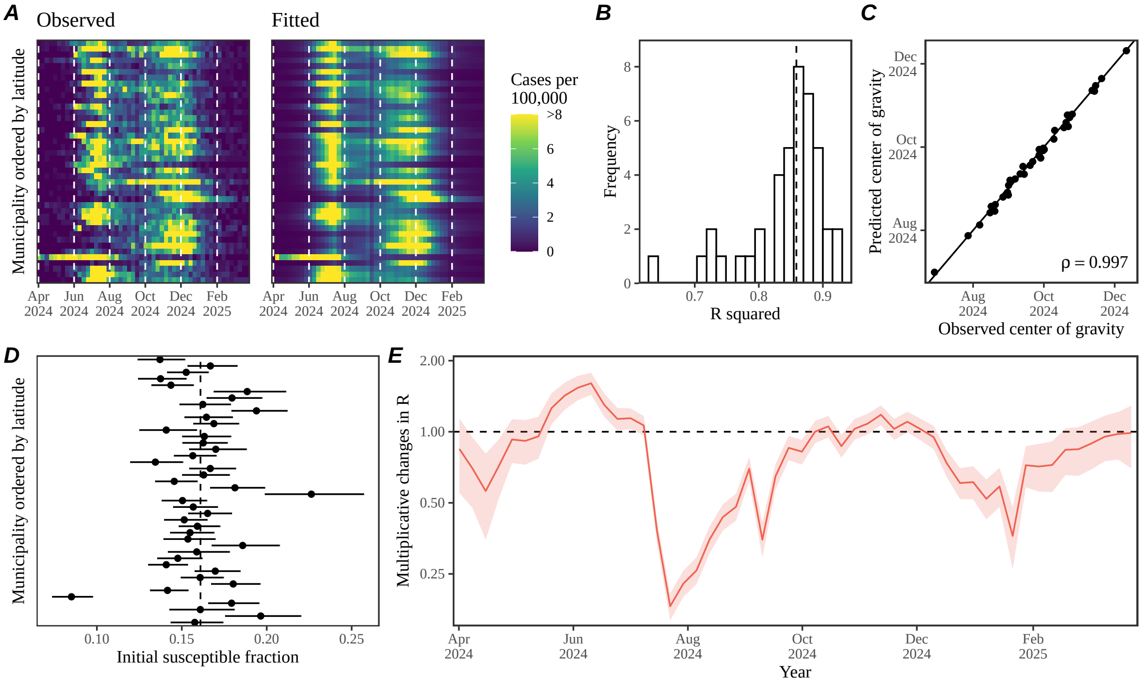

Figure S5: **Alternative SEIR model fits assuming variation in initial susceptible fraction.** (A) Comparisons between the observed and predicted epidemic dynamics across municipalities with more than 400 total cases, ordered by latitude. (B) The bar plot represents the distribution of R squared values for model fits. The vertical dashed line represents the median. (C) Relationship between the estimated and predicted center of gravity. The solid line indicated the one-to-one relationship. (D) Estimated initial susceptible fraction $S(0)$ across municipalities, ordered by latitude. Points and error bars represent posterior medians and corresponding 95% credible intervals. (E) Estimated time-varying pertussis transmission multiplier. The red line and shaded regions represent the posterior median and the corresponding 95% credible interval.

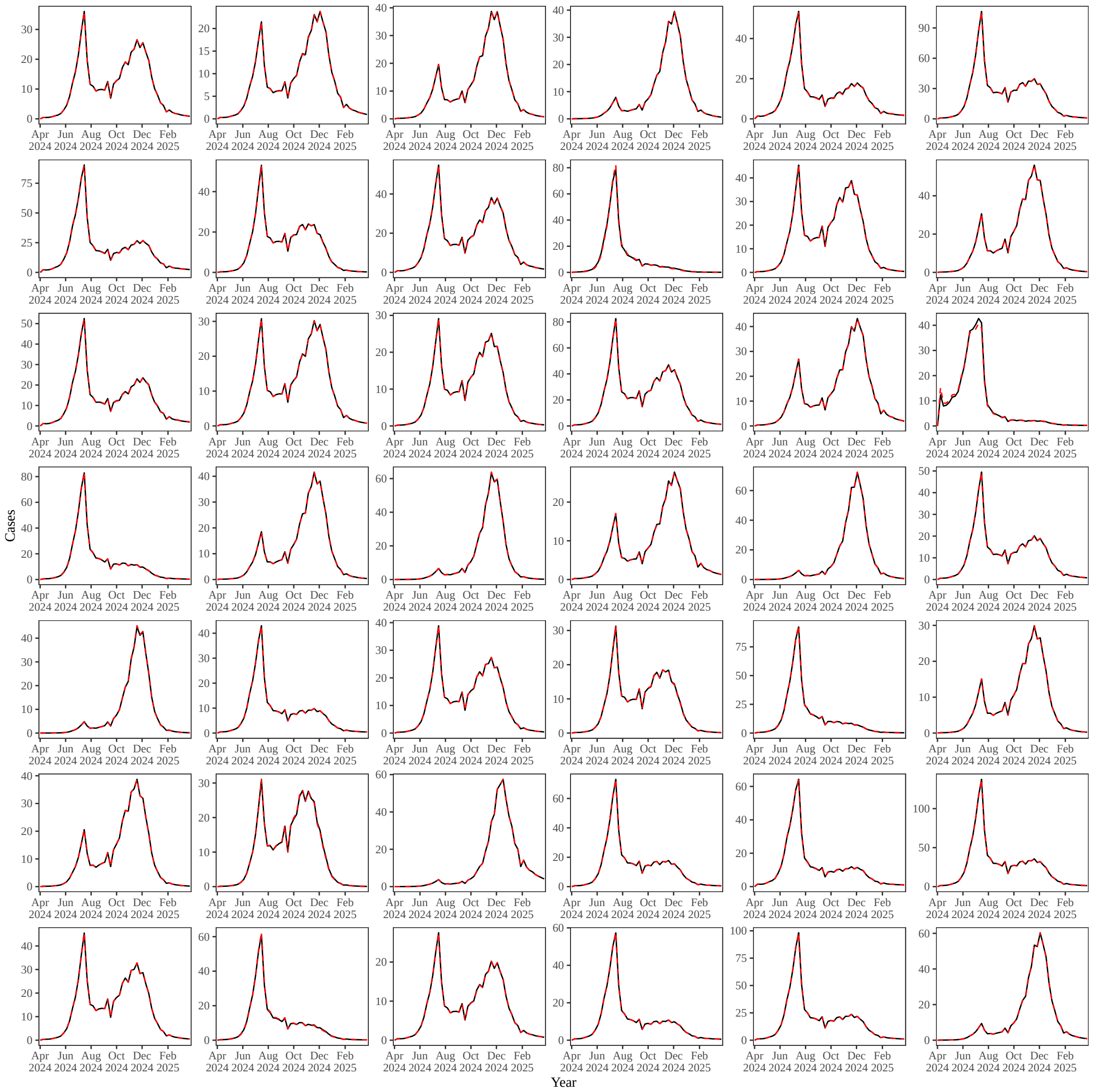

Figure S6: **Comparisons between model fits across two different models.** Solid lines represent posterior median predictions based on the model assuming separate $\mathcal{R}_0$ in each region. Red lines represent posterior median predictions based on the model assuming separate $S(0)$ in each region.

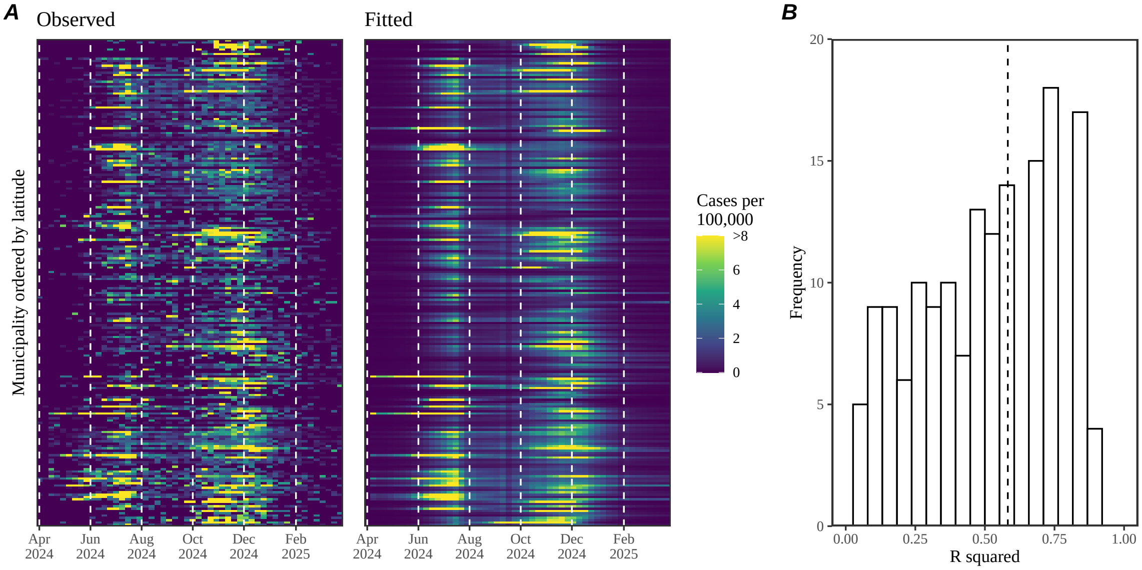

Figure S7: **Validation of the alternative transmission model using time series data from municipalities with less than 400 total cases.** (A) Comparisons between the observed and predicted epidemic dynamics across municipalities with less than 400 total cases, ordered by latitude. (B) The bar plot represents the distribution of R squared values for model fits. The vertical dashed line represents the median. Initial infected, initial susceptible, and reporting probability were estimated for each region while assuming a known transmission variation term $\delta(t)$ and basic reproduction number.

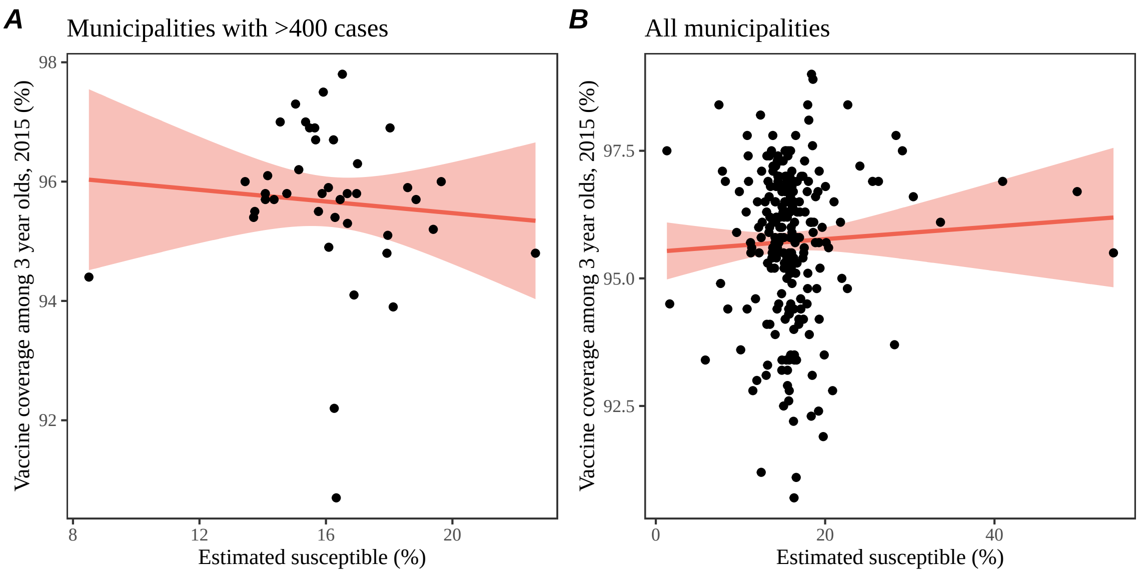

Figure S8: **Relationship between estimated initial susceptible and vaccine coverage for (A) municipalities with** $>$ 400 **cases and (B) for all municipalities.** Each point represents the estimate initial susceptible and vaccine coverage value for each municipality. Red lines and shaded regions represent the linear regression fit and corresponding 95% confidence intervals.

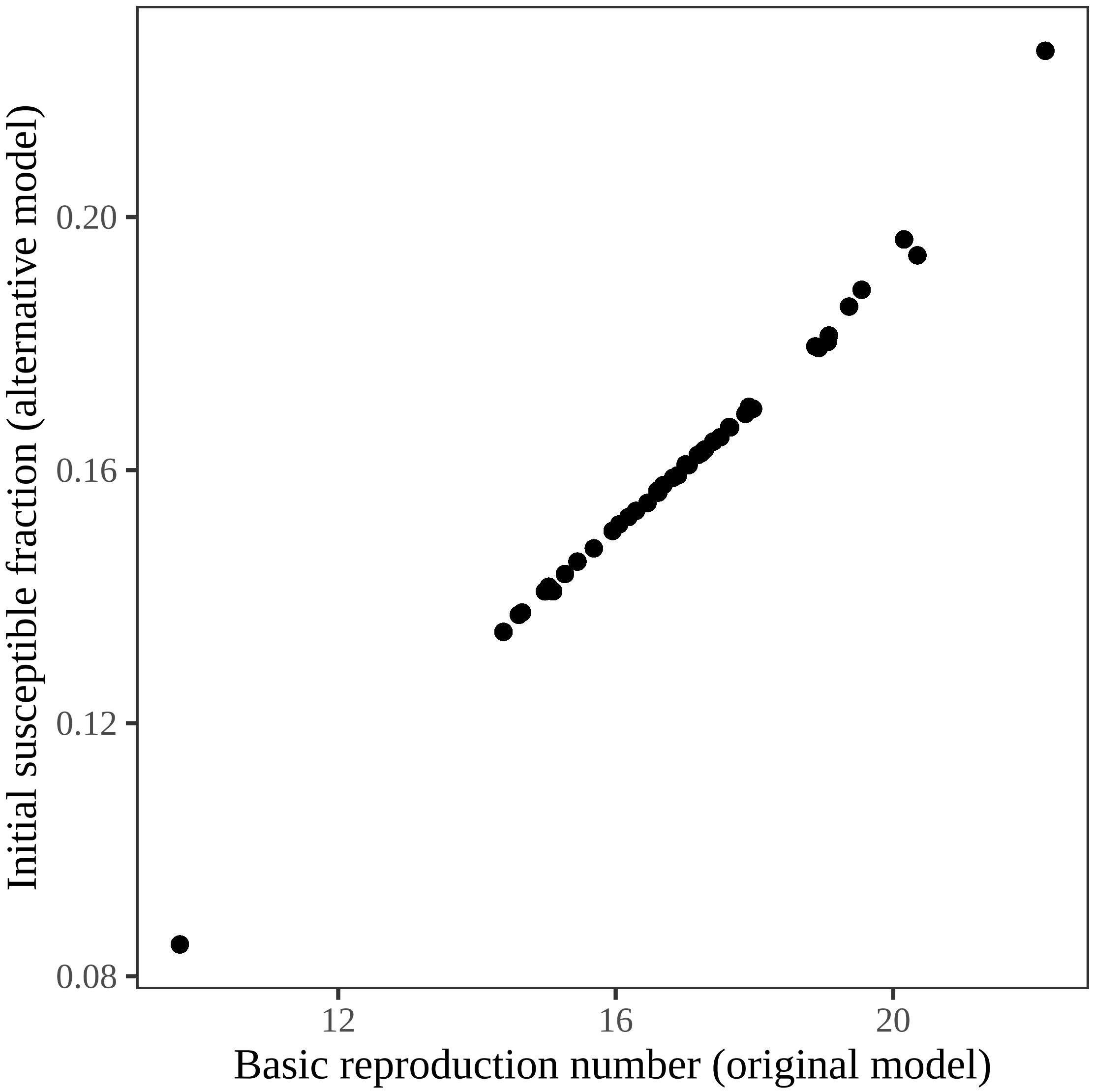

Figure S9: **Comparisons between estimated basic reproduction number and initial susceptible fraction across two different models.** Each point represents the estimated posterior median for the basic reproduction number $\mathcal{R}_0$ and initial susceptible fraction $S(0)$ from two separate models in each region.

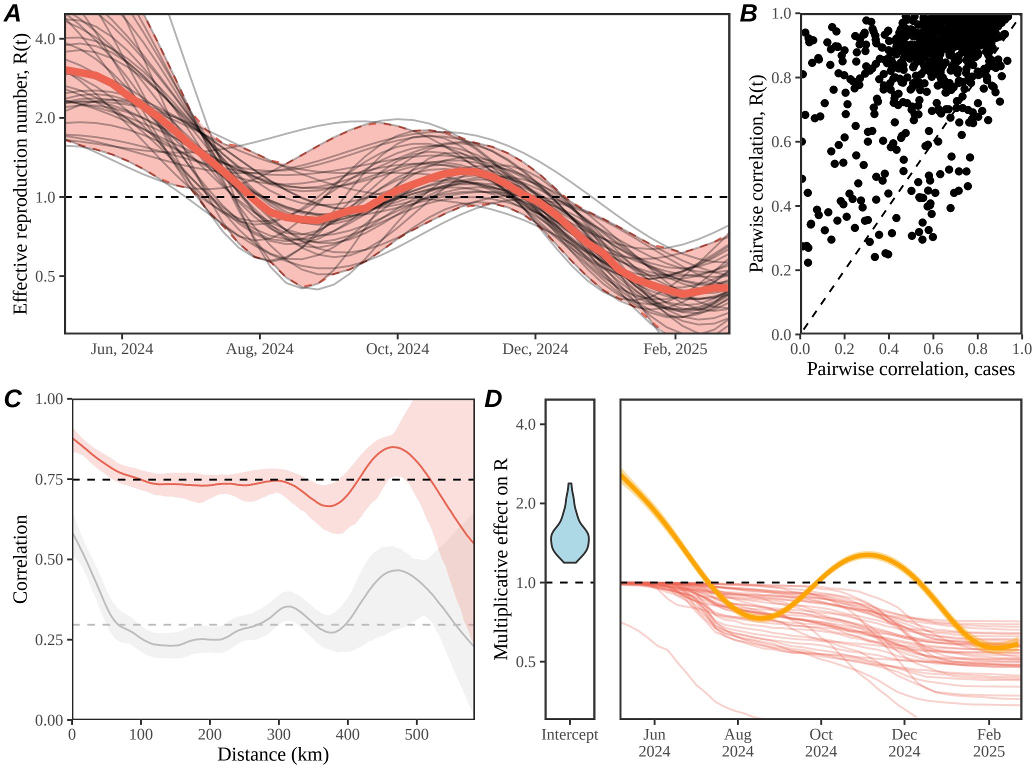

Figure S10: **Sensitivity analysis of spatial variation in the estimates of effective reproduction number assuming longer serial intervals.** (A) Each black line represents the estimated effective reproduction number for a given municipality with more than 400 total cases. The red solid line and shaded regions represent the median and 95% quantiles of effective reproduction number estimates. (B) Relationship between correlation coefficient for $\mathcal{R}(t)$ and correlation coefficient for reported cases across all pairwise municipality combinations with more than 400 total cases. (C) Spatial synchrony in $\mathcal{R}(t)$ (red) versus logged cases (black) as a function of distance. The solid line and shaded regions represent the median estimate and the corresponding 95% confidence interval. The dashed horizontal line represents the mean correlation. (D) Estimated multiplicative effects on $\mathcal{R}(t)$ from the regression analysis. The blue shaded violin represents the distribution of intercept effect, where a separate intercept term is estimated for each municipality. The red lines represent the estimated susceptible depletion effect for each municipality. The orange line and shaded regions represent the estimated changes in transmission from a smooth term and the corresponding 95% confidence interval.

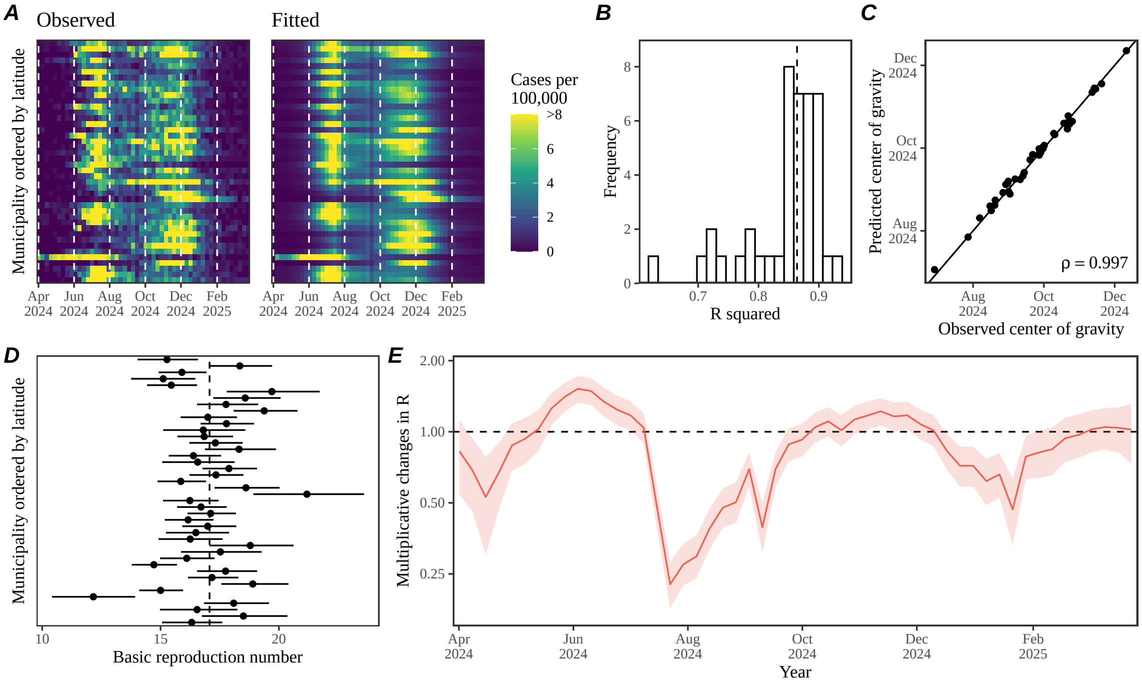

Figure S11: **SEIR model fits to spatiotemporal pertussis epidemic data assuming negative binomial observation error.** (A) Comparisons between the observed and predicted epidemic dynamics across municipalities with more than 400 total cases, ordered by latitude. (B) The bar plot represents the distribution of R squared values for model fits. The vertical dashed line represents the median. (C) Relationship between the estimated and predicted center of gravity. The solid line indicates the one-to-one relationship. (D) Estimated basic reproduction number $\mathcal{R}_0$ across municipalities, ordered by latitude. Points and error bars represent posterior medians and corresponding 95% credible intervals. (E) Estimated time-varying pertussis transmission multiplier. The red line and shaded regions represent the posterior median and the corresponding 95% credible interval.

References

[1] Aaron A King, Dao Nguyen, and Edward L Ionides. Statistical inference for partially observed Markov processes via the R package pomp. *Journal of Statistical Software*, 69:1–43, 2016.
